# The association of pain and stiffness with fatigue in incident polymyalgia rheumatica: baseline results from the polymyalgia rheumatica cohort study

**DOI:** 10.1017/S1463423619000082

**Published:** 2019-05-14

**Authors:** J.A. Prior, S. Muller, T. Helliwell, S.L. Hider, K. Barraclough, B. Dasgupta, C.D. Mallen

**Affiliations:** 1 Research Institute for Primary Care and Health Sciences, Keele University, Staffordshire, UK; 2 Haywood Academic Rheumatology Centre, Haywood Hospital, Stoke-on-Trent, Staffordshire, UK; 3 Painswick Surgery, Gloucestershire, UK; 4 Southend University Hospital, Westcliff-on-Sea, Essex, UK

**Keywords:** fatigue, pain, polymyalgia rheumatica, primary care, stiffness

## Abstract

We aimed to examine the association between pain, stiffness and fatigue in newly diagnosed polymyalgia rheumatica (PMR) patients using baseline data from a prospective cohort study. Fatigue is a known, but often ignored symptom of PMR. Newly diagnosed PMR patients were recruited from general practice and mailed a baseline questionnaire. This included a numerical rating scale for pain and stiffness severity, manikins identifying locations of pain and stiffness and the FACIT-Fatigue questionnaire. A total of 652 PMR patients responded (88.5%). The mean age of responders was 72.6 years (SD 9.0) and the majority were female (62.0%). Manikin data demonstrated that bilateral shoulder and hip pain and stiffness were common. The mean fatigue score (FACIT) was 33.9 (SD 12.4). Adjusted regression analysis demonstrated that a higher number of pain sites (23–44 sites) and higher pain and stiffness severity were associated with greater levels of fatigue. In newly diagnosed PMR patients, fatigue was associated with PMR symptom severity.

## Introduction

Polymyalgia rheumatica (PMR) is an inflammatory condition characterised by bilateral pain and stiffness in the shoulder and hip girdles. In the UK, PMR is the most prevalent inflammatory rheumatic disease in adults aged 50 years and over, peaking in incidence in those aged 70–79 years (22.9 per 10 000 person-years) (Smeeth, Cook, and Hall, 2006).

Patients with PMR have identified fatigue to be an important symptom and outcome measure for clinical research (Helliwell *et al*., 2016), and despite some patients reporting this to be more troublesome than the pain and stiffness which often characterises their condition (Mackie *et al*., 2015), fatigue is often neglected and frequently not explored, thus remaining poorly characterised (Helliwell *et al*., 2016). The experience of fatigue in other inflammatory conditions, such as rheumatoid arthritis (RA), suggests it is common and is associated with several condition-specific factors, including levels of pain and stiffness (Nikolaus *et al*., 2013). As such, fatigue is included as a core outcome measure for patients with RA (Kirwan *et al*., 2007), but the current evidence describing the presence of fatigue in PMR patients is mixed (Chuang *et al*., 1982; Green *et al*., 2014; Levy *et al*., 2015) and has not examined the role of key associated symptoms such as pain and stiffness.

The general role of pain and stiffness, the cardinal symptoms of PMR, on fatigue in these patients’ remains unclear. Examining these associations at initial PMR diagnosis in primary care, where the majority of these patients receive their care, will allow us to better understand and characterise the early stages of PMR. Such information may also highlight patients who would benefit from additional or more prompt interventions at the early stages of disease. Our aim was to describe how pain and stiffness at diagnosis were related to fatigue in PMR patients in primary care.

## Materials and methods

### Study design and population

This study used baseline data from a prospective observational inception cohort of PMR patients recruited in UK primary care (Muller *et al*., 2012). Patients aged 18 years and older were recruited at the time of PMR diagnosis from 382 research-active general practices across England. Recording a new diagnosis of PMR in the patient’s electronic record triggered a template, which provided a summary of the British Society of Rheumatology PMR diagnostic process and prompted the GP to request recommended clinical investigations and recruit the patient into the study. Patients were mailed a postal questionnaire and consent form by the research team.

Responders to the baseline survey were followed-up at six further time points over two years. This study presents data from the baseline phase of the study and approval was obtained from the Staffordshire Local Research Ethics Committee (Ref no.: 12/WM/0021).

### Outcome measures

**Figure 1:**
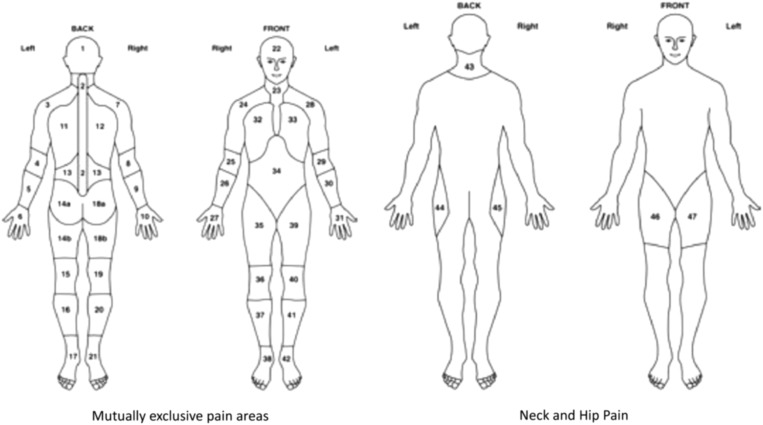
Pain and stiffness manikins: definition of hips and shoulders. Shoulders are defined as areas 3, 7, 24 and 28. Hips are defined as areas 44, 45, 46 and 47 (Birrell *et al*. 2000)

Anatomical location of pain and stiffness were elicited using body manikins, up to a maximum of 44 body areas (Figure 1) (Muller *et al*., 2012). In addition to manikin data, participants were asked to rate the severity of their pain and stiffness using two separate 0–10 numerical rating scale (NRS), with 0 indicating no pain/stiffness and 10 the worst pain/stiffness imaginable. Participants were also asked whether they had difficulty raising their arms above their head.

Fatigue was measured using the FACIT-Fatigue questionnaire, which has previously been validated for use in other conditions, including psoriatic arthritis (Yellen *et al*., 1997; Chandran *et al*., 2007). Scores range from 0 to 52; lower scores indicate more fatigue, with normative US general population data (*n*=1,075) reporting a mean score of 40.1 (SD 10.4) (Cella, 2012). A minimal clinically important difference (MCID) of three points is recommended to determine a cross-sectional difference between groups (Cella *et al*., 2002).

Other survey outcome measures recorded sleep problems (insomnia severity index (ISI) (Morin, 1993), anxiety [generalised anxiety disorder (GAD-7)] (Spitzer *et al*., 2006) and depression [patient health questionnaire (PHQ-8)] (Kroenke *et al*., 2009) within the last two weeks. The baseline questionnaire also collected data on age, gender, body mass index (BMI) (calculated from self-reported weight and height) and deprivation status (indices of multiple deprivation (IMD)] (Communities and Neighbourhoods, 2011).

### Statistical analysis

The characteristics of the study sample were initially summarised using descriptive statistics. The mean age (SD) and gender were reported, and self-report BMI was categorised into four groups (<25.0 healthy weight, 25.0–29.9 overweight, 30.0–34.9 obese and ⩾35.0 severely obese) and IMD was categorised into three groups (the 20% least deprived, mid-deprived and 20% most deprived). The total number of pain/stiffness sites for each participant was reported in quartiles (pain: 0–9, 10–15, 16–22, 23–44 sites; stiffness: 0–5, 6–11, 12–19, 20–44 sites). The manikin data were also used to dichotomise participants into those who had experienced bilateral pain/stiffness in the shoulder/hips using relevant manikin locations [shoulder areas: either locations 3 or 28 (left) and 7 or 24 (right); hip areas: either 44 or 47 (left) and 45 or 46 (right)] (Muller *et al*., 2012) (Figure 1). Pain and stiffness severity scores were dichotomised at the median [low (0–7) versus high (8–10)] in order to make results more clinically meaningful.

Mean scores (SD) were generated for the FACIT-Fatigue questionnaire. Scoring of the ISI creates four severity categories: 0–7, no clinically significant insomnia; 8–14, sub-threshold insomnia; 15–21, clinical insomnia (moderate severity) and 22–28, clinical insomnia (severe). GAD-7 scores were categorised into those with no anxiety (0–4 points), mild (5–9), moderate (10–14) and severe anxiety (15–21) and PHQ-8 scores into no depression (0–4), mild (5–9), moderate (10–14), moderately severe (15–19) and severe depression (20–24) using already existing scoring thresholds (Spitzer *et al*., 2006; Kroenke *et al*., 2009). The mean FACIT-Fatigue scores were compared across these socio-demographic and health-related factors using analysis of variance.

Linear regression was performed using Stata 14 (StataCorp., 2015) to assess the association of pain and stiffness characteristics with FACIT-Fatigue score. Results were reported as regression coefficients with 95% confidence interval (95% CI), first crude associations were presented and then results were adjusted for age, gender, deprivation status, BMI, anxiety, depression and insomnia.

## Results

### Sample characteristics

A total of 652 patients responded to the baseline questionnaire (adjusted response rate 88.5%). The mean age of responders was 72.6 years (SD 9.0) and the majority were female (62.0%). As previously described, responders were similar to non-responders in terms of age and gender, although non-responders were more likely to be more deprived (Muller *et al*., 2016), wherein 13.1% reported symptoms of anxiety and 21.8% reported symptoms of depression (GAD-7 and PHQ-8 scores >10, respectively). Nearly a quarter of respondents with newly diagnosed PMR experienced clinically significant insomnia (23.6%) (Table 1).

**Table 1:** PMR cohort characteristics (*n*=652)

Characteristic	*n* (%)
Age [Mean (SD)]	72.6 (9.0)
Age (Year categories)	
⩽64	128 (17.4)
65–69	125 (16.7)
70–74	148 (20.1)
75–80	162 (22.0)
⩾80	173 (23.5)
Female gender (%)	457 (62.0)
Deprivation status	
Least deprived (20%)	125 (19.8)
Middle (60%)	389 (61.5)
Most deprived (20%)	119 (18.7)
Body mass index	
<25 kg/m^2^	210 (33.8)
25.0–29.9 kg/m^2^	251 (40.4)
30.0–34.9 kg/m^2^	99 (16.0)
⩾35 kg/m^2^	61 (9.8)
Anxiety (GAD-7) (*n*, %)	
None (<5)	397 (65.2)
Mild (5–9)	132 (21.7)
Moderate (10–14)	49 (8.0)
Severe (15>)	31 (5.1)
Depression (PHQ-8) (*n*, %)	
None (0–4)	318 (52.9)
Mild (5–9)	152 (25.3)
Moderate (10–14)	70 (11.7)
Moderately severe (15–19)	38 (6.3)
Severe (20>)	23 (3.8)
Insomnia (ISI) (*n*, %)	
No clinically significant insomnia	243 (39.6)
Subthreshold insomnia	226 (36.8)
Clinical insomnia (moderate severity)	112 (18.2)
Clinical insomnia (severe)	33 (5.4)
Can you raise arms above head?	
Yes	184 (28.6)
No	413 (64.2)
Don’t know	46 (7.2)

The mean FACIT-Fatigue score was 33.9 (SD 12.4), and did not significantly vary by age or deprivation tertile. However, levels of fatigue were greater in women and in those with higher BMI, and higher levels of anxiety and depression (Table 2). Bilateral shoulder (87.3%) and hip (63.5%) pain were common. Nearly half of patients experienced pain in ⩾16 separate body locations (47%) and stiffness at ⩾12 discrete body sites (47.2%); 71.7% reported bilateral shoulder stiffness and 52.0% had bilateral hip stiffness (Table 3).

**Table 2:** FACIT-fatigue score by sample characteristics

Characteristic	FACIT-Fatigue Mean (SD)	*P*-value
PMR cohort	33.9 (12.4)	–
Gender		
Female	32.0 (12.9)	<0.001
Male	37.0 (10.8)	
Age		
⩽64	32.2 (12.6)	0.29
65–69	33.4 (13.3)	
70–74	35.7 (12.4)	
75–79	34.0 (12.2)	
⩾80	34.0 (11.5)	
Deprivation		
Least deprived (20%)	33.6 (12.9)	0.35
Middle (60%)	34.3 (12.2)	
Most deprived (20%)	32.4 (12.7)	
BMI		
<25 kg/m^2^	35.5 (11.8)	<0.001
25.0–29.9 kg/m^2^	35.0 (12.1)	
30.0–34.9 kg/m^2^	31.4 (12.5)	
⩾35 kg/m^2^	27.6 (12.8)	
Anxiety		
None	38.5 (9.8)	<0.001
Mild	29.2 (11.1)	
Moderate	23.4 (12.5)	
Severe	15.5 (8.6)	
Depression		
None	41.8 (7.3)	<0.001
Mild	30.9 (9.3)	
Moderate	22.1 (9.2)	
Moderately severe	16.8 (6.3)	
Severe	12.8 (7.4)

**Table 3:** Association between experiences of pain and stiffness characteristics and fatigue

		FACIT-Fatigue score	
		Linear regression (95% confidence interval)	
		Unadjusted	Adjusted
	*n* (%)		Age, gender, deprivation, BMI, anxiety, depression and insomnia
*Pain*			
Pain rating			
Low (0–7)	217 (33.9)	0	0
High (8–10)	423 (66.1)	**−3.76** (**−5.8 to −1.7)**	**−1.66** (**−3.1 to −0.2)**
No of pain sites			
0–9	180 (27.6)	0	0
10–15	166 (25.5)	−1.00 (−3.6 to 1.62)	−0.81 (−2.7 to 1.0)
16–22	143 (21.9)	**−3.77** (**−6.5 to −1.0)**	−1.10 (−3.1 to 0.8)
23–44	163 (25.0)	**−7.12** (**−9.7 to −4.5)**	**−3.45** (**−5.3 to −1.6)**
Bilateral shoulder pain			
No	83 (12.7)	0	0
Yes	569 (87.3)	−2.83 (−5.8 to 0.19)	−1.02 (−3.2 to 1.1)
Bilateral hip pain			
No	238 (36.5)	0	0
Yes	414 (63.5)	−1.37 (−3.4 to 0.7)	−1.44 (−2.9 to 0.0)
*Stiffness*			
Stiffness rating			
Low (0–7)	255 (39.7)	0	0
High (8–10)	387 (60.3)	**−3.55** (**−5.5 to −1.6)**	**−1.48** (**−2.9 to −0.1)**
No of stiffness sites			
0–5	181 (27.8)	0	0
6–11	164 (25.1)	−2.15 (−4.8 to 0.5)	−0.30 (−2.2 to 1.6)
12–19	157 (24.1)	−1.69 (− 4.4 to 0.1)	0.23 (−1.7 to 2.2)
20–44	150 (23.0)	**−5.96** (**−8.7 to −3.3)**	−1.62 (−3.5 to 0.3)
Bilateral shoulder stiffness			
No	184 (28.2)	0	0
Yes	468 (71.8)	**−2.86** (**−5.0 to −0.7)**	−0.41 (−2.0 to 1.2)
Bilateral hip stiffness			
No	313 (48.0)	0	0
Yes	339 (52.0)	−1.58 (−3.5 to 0.4)	−0.49 (−1.9 to 0.9)
Can you raise arms above your head?			
Yes	184 (30.8)	0	0
No	413 (69.2)	**−2.35** (**−4.1 to −0.6)**	−0.38 (−1.6 to 0.9)

Higher FACIT-Fatigue score is indicative of lower fatigue; therefore, a negative regression coefficient represents a higher level of fatigue. A different in score of three points is considered clinically relevant [13].

Bold values indicates statistically significant.

### Association between pain and fatigue

Mean fatigue scores were lower (ie more fatigue) in those reporting a pain severity of ⩾8 on the 0–10 NRS compared to those reporting a score of ⩽7 [regression coefficient (95% CI): –3.76 (−5.8 to –1.7)]. After adjustment for age, gender, deprivation status, BMI, anxiety, depression and insomnia the association was reduced, but still statistically significant [−1.66 (−3.1 to –0.2)] (Table 3).

Unadjusted analysis demonstrated an association between a higher number of pain sites and greater fatigue. However, after adjustment only the participants with the highest quartile of pain sites (23–44 sites) remained significantly different [−3.45 (−5.3 to –1.6)] from the group with the lowest number of pain sites (0–9 sites). No association was demonstrated between fatigue and reporting either bilateral shoulder [−1.02 (−3.2 to 1.1)] or hip pain [−1.44 (−2.9 to −0.0)].

### Association between stiffness and fatigue

In the unadjusted analysis, higher stiffness severity (⩾8) was associated with experiencing significantly greater fatigue [−3.55 (−5.5 to –1.6)]. Though attenuated, this remained a statistically significant association after adjustment [−1.48 (−2.9 to –0.1)] and was the only stiffness characteristic to be associated with fatigue after adjustment.

## Discussion

Pain and stiffness were common in this primary care cohort of newly diagnosed PMR patients. Those reporting higher levels of pain and stiffness had significantly greater levels of fatigue than those with a lower symptom severity. Furthermore, those reporting pain at a higher number of anatomical sites, rather than just isolated to the classical shoulder and hip regions reported the greatest experience of fatigue.

The mean FACIT-Fatigue score for our PMR cohort [33.9 (SD 12.4)] was lower than that of a similarly aged sample (65 years or older) from the US general population (*n*=164, mean 38.6, SD 10.8) (Cella, 2012), demonstrating a greater level of fatigue experienced by PMR patients than the general population. Though high levels of pain and stiffness severity were statistically associated with an increased experience of fatigue, differences between groups were smaller than the three-point MCIDs suggested as being clinically important. Only those who reported pain in more than 23 body sites experienced such a clinically meaningful difference in fatigue score compared to those with 0–9 sites of pain. This highlights that for these participants in particular, there is a relationship between pain and fatigue, though we are unable to determine the contribution of other comorbidities that may account for pain in some others areas of the body.

Compared to other inflammatory conditions, our cohort reported more fatigue than US patients with newly diagnosed ulcerative colitis [mean FACIT-Fatigue score 39.4 (SD) (10.6)] and psoriatic arthritis [35.8 (SD 12.4)] (Chandran *et al*., 2007). However, our sample reported less fatigue than a sample of US RA patients (mean age 52) and a sample of ankylosing spondylitis patients (mean age 42), who had mean FACIT-fatigue scores of 29 and 24, respectively (Revicki *et al*., 2011; Strand *et al*., 2012). Though such variation in fatigue may be related to heterogeneity of study design (these previous studies were secondary care samples and clinical trials), the level of fatigue reported by our cohort appears comparable to other inflammatory conditions and worse than that experienced by the general older age population. However, further research is needed to determine the impact of fatigue in patients with PMR, whether fatigue improves in parallel with other clinical symptoms and the optimal ways to address fatigue in clinical practice.

Apart from those patients with PMR reporting the highest stiffness ratings, fatigue was not associated with the general number of stiff sites or stiffness at specific locations. Though stiffness is clearly a common symptom in PMR and will cause difficulties in multiple aspects of daily living and functioning (Mackie *et al*., 2015), it did not seem to correlate with fatigue reporting in this newly diagnosed PMR cohort.

Individuals newly diagnosed with PMR who report fatigue as a significant problem may benefit from modified pain treatment or management that directly addresses these issues. A randomised controlled trial conducted by Durcan *et al.* found that pain, stiffness, sleep quality and fatigue all significantly improved in RA patients prescribed a 12-week home-based exercise intervention (Durcan, Wilson, and Cunnane, 2014). Such interventions may also modify factors like anxiety and depression, which are also likely to influence the experience of fatigue.

This prospective cohort is the first study of patients newly diagnosed with PMR in primary care. Such a sample is representative of patients with PMR, as the majority are managed in primary care (Dasgupta *et al*., 2010). Furthermore, response bias was minimised due to high number of returned questionnaires (88.5%). Although it is possible that some people referred into the study may not have been considered to have PMR if examined by a rheumatology specialist, we consider our sample representative of those diagnosed and treated as having PMR in UK primary care. The provision of information to GPs through the recruitment template, the age/gender balance of our sample and the large proportions with bilateral shoulder and/or hip pain and stiffness adds further confidence of accuracy of PMR diagnosis, rather than alternative conditions for which widespread pain and fatigue are also symptoms, for example fibromyalgia.

## Conclusions

In conclusion, severe pain was predominantly associated with fatigue in newly diagnosed PMR patients, but it is those patients who have pain in a high number of body sites who experience fatigue that is clinically meaningful. Fatigue is of concern to patients’ with PMR, but often under-represented in research and not prioritised by clinicians. Future studies should characterise the experience of fatigue over-time in patients’ with PMR and determine how this relates to reported pain and stiffness. This may highlight those who warrant earlier or more specific interventions.

## Authors’ contributions

Guarantor of overall study integrity: S.M. and C.D.M. Study concept and design: S.M., B.D., K.B., T.H., S.H., S.L., C.D.M. Data collection and interpretation: J.A.P., S.M., S.L. and C.D.M. Statistical analysis: J.A.P. and S.M. Manuscript preparation: J.A.P., S.M., T.H., S.H., S.L., K.B., B.D. and C.D.M. Final approval of manuscript: J.A.P., S.M., T.H., S.H., S.L., K.B., B.D. and C.D.M.
